# Ethical implications of economic compensation for voluntary medical male circumcision for HIV prevention and epidemic control

**DOI:** 10.1371/journal.pgph.0001361

**Published:** 2022-12-16

**Authors:** Johannes Köhler, Jerome Amir Singh, Rennie Stuart, Julia Samuelson, Andreas Alois Reis

**Affiliations:** 1 Department of Anesthesiology and Critical Care Medicine, Kantonsspital Münsterlingen, Münsterlingen, Switzerland; 2 School of Law, University of KwaZulu-Natal, Durban, South Africa; 3 Dalla Lana School of Public Health, University of Toronto, Toronto, Canada; 4 UNC Bioethics Center, Department of Social Medicine, University of North Carolina at Chapel Hill, Chapel Hill, North Carolina, United States of America; 5 Global Programmes on HIV, Hepatitis, STIs, World Health Organization, Geneva, Switzerland; 6 Health Ethics and Governance Unit, World Health Organization, Geneva, Switzerland; PLOS: Public Library of Science, UNITED STATES

## Abstract

Despite tremendous efforts in fighting HIV over the last decades, the estimated annual number of new infections is still a staggering 1.5 million. There is evidence that voluntary medical male circumcision (VMMC) provides protection against men’s heterosexual acquisition of HIV-1 infection. Despite good progress, most countries implementing VMMC for HIV prevention programmes are challenged to reach VMMC coverage rates of 90%. Particularly for men older than 25 years, a low uptake has been reported. Consequently, there is a need to identify, study and implement interventions that could increase the uptake of VMMC. Loss of income and incurred transportation costs have been reported as major barriers to uptake of VMMC. In response, it has been suggested to use economic compensation in order to increase VMMC uptake. In this discussion paper, we present and review relevant arguments and concerns to inform decision-makers about the ethical implications of using economic compensation, and to provide a comprehensive basis for policy and project-related discussions and decisions.

## Introduction

Although an increasing number of people living with HIV gain access to antiretroviral therapy, the estimated annual number of new infections remains disconcerting with 1.5 million new infections globally in 2020 [1]. There is robust evidence that *voluntary medical male circumcision* (VMMC) provides protection against men’s heterosexual acquisition of HIV-1 infection, reducing their risk by about 60% [2–5]. According to the Copenhagen Consensus, circumcising 90% of HIV-negative men in the five worst-affected countries is one of the 19 best investments to achieve the post-2015 development agenda, with a benefit of US$ 28 for every dollar invested and averting 1.1m infections by 2030 [6]. UNAIDS and the World Health Organization (WHO) have indicated that reaching the 2020 and 2030 Fast Track prevention goals will be impossible without the VMMC intervention in HIV prevention programmes in priority countries in eastern and southern Africa, as achieving only treatment scale-up targets will most likely not be sufficient [7]. Despite good progress, most countries (except Kenya) are challenged to reach VMMC coverage rates of 90% among males 15–49 years; particularly for men older than 25–30 years, a low uptake has been reported [8–10].

Consequently, there is a need to identify, study and implement interventions that increase the uptake of VMMC, especially in eastern and southern Africa. In between the extremes of forcing people to adopt certain behaviours (e. g., to wear seatbelts) or rendering a specific choice significantly more costly (e. g., taxation of tobacco) on the one hand, and merely informing and rationally persuading them (e. g., providing information on the harms associated with obesity) on the other hand, alternative strategies have been proposed: the use of *nudges* and *incentives*. Although there is no consensus on the definition of nudging, it generally describes a strategy of influencing “behaviour in a predictable way without forbidding any previously available courses of actions or making alternatives appreciably more costly in terms of time, trouble, social sanctions, and so forth” [11]. Characteristically, *nudges* “are called for because of flaws in individual decision-making, and they work by making use of those flaws” [12]. Whereas nudges must not “impose significant material incentives” [13], *incentives* work by plainly offering the targeted subject some monetary or non-monetary benefit. In health, incentives have been implemented in different contexts ranging from financial incentives for weight loss offered by companies to their employees [14] to extensive governmental programmes such as Progresa/Oportunidades in Mexico [15]. In the field of HIV, incentives have been successfully used to reduce risky behaviours, for example, cash transfer contingent on negative STI tests [16], to boost HIV testing [17], or to increase viral suppression and clinic attendance among HIV-positive patients in care [18].

Concerns about lost income and transportation cost, requiring out-of-pocket expenditures, are a commonly reported barrier to access VMMC [19–21]. To address this issue, a strategy of economic compensation has been tested in several countries: providing food or other types of vouchers—conditional on seeking VMMC services or becoming circumcised—were found to be effective in increasing VMCC uptake among 25- to 49-year-old men [22]. We will argue that while economic compensations may help reduce inequities in access to prevention services, their usage also raises a host of ethical issues that warrant close scrutiny. When planning or implementing VMMC programmes, countries need to address significant ethical concerns about circumcision itself and its use in public health [23–25]. Here, however, we do not discuss the general ethical implications of circumcising men for prevention, but instead, focus our analysis on specific ethical considerations regarding the *use of economic compensation* to men for undergoing such a procedure. Even though we discuss the specific case of economic compensations in VMMC, the general considerations and implications presented hold true for the use of such compensations in other health interventions. In the following discussion paper, we present and review relevant arguments and concerns. We aim to inform decision-makers about the ethical implications of using economic compensations and provide a comprehensive basis for policy and project-related discussions and decisions.

### Economic compensation for VMMC

While ‘economic compensation’ can easily be defined and quantified, ‘economic loss’ is much harder to grasp. For the purpose of this work, we define economic loss as **“***the amount of money or non-monetary goods lost to the subject due to undergoing VMMC*.*”* However, actually coming up with specific amounts is remarkably difficult. To calculate them, we would likely consider the economic loss suffered by the participant while the procedure is being performed (which could be measured in hours, including transit and waiting times) and transportation cost. Yet, this is where the problems start: On what basis should the lost wage be calculated? Minimum wage? Average wage? What about participants with highly fluctuating wages (day labourers, street vendors, taxi drivers)? Equal difficulties apply to transportation costs. Also, must the compensation comprise the (average?) healing period? Should it also entail compensation for personal suffering due to the procedure? The elusive nature of “economic loss”—and the difficulty to come up with a coherent and universal calculation—should be kept in mind for the following discussion.

### Reimbursement of the economic loss and incentivization

Considering economic compensation (C) and economic loss (L) in the case of VMMC, we encounter two pivotal scenarios: (1) the compensation received is smaller or equal to the economic loss (*C* ≤ *L*) or (2) the compensation is greater than the economic loss (*C* > *L*). It could be argued that whenever C = L, the reimbursement of economic loss is a simple economic transaction without much ethical concern; the same might be true for C < L, although it is obviously an imperfect compensation and might constitute a bad bargain (and consequently uptake might be low). Obviously, there is no economic benefit for *C*–*L* ≤ 0. However, we argue that this line of thinking is wrong for two reasons.

First, ensuring C = L will be nearly impossible in any real-life implementation. Any *default* compensation will be above the economic loss (C > L) for some subjects and at the same time insufficient (C < L) for others. For instance, paying half a week’s minimum wage might constitute a significant incentive for someone currently out of work, but the same amount does not even come close to compensating someone earning many times the minimum wage. Even with *individually* calculated economic compensations (keep in mind how hard it is to determine the actual economic loss in the first place)—presumably not feasible for practical reasons—, the risk of paying more than required due to miscalculation, or intentional misdeclaration by participants (wages, transportation, healing period, …) remains. Interestingly, the phenomenon of the diminishing marginal utility of money does not hold in cases of severe poverty because (economic) desperation makes even small incentives critical. In summary, it seems highly unlikely that paying economic compensations, however small, will not result in incentivization—at least for some in the targeted population. Hence, the ethical implications of incentives have to be taken into account when paying compensations.

Second, all—even small—economic compensations can have effects that go beyond mere economic rationality. Among others, concerns about personal responsibility, the creation of expectations, resource allocation and justice have been voiced.

### Incentivisation and autonomy

In many countries, medicine and public health have gone through a crucial transformation in the last century: away from a paternalistic, ‘your-doctor-knows-best’ attitude to a more patient-centred, autonomy enhancing, collaborative relationship. If we value the capacity of people to make their own decisions, free from undue external influence, we might fear that an incentive corrupts their autonomy; particularly when we consider the compensation offered to the participant too high in proportion to the participant’s income and economic situation, and unlikely to be easily declined by the participant. A similar concern is discussed in research ethics, where such substantial compensation has been termed “undue inducement” [26, 27]. Saghai proposes a necessary condition for the preservation of freedom of choice: *A’s influence to get B to do X is substantially non-controlling when B could easily not do X if she did not want to do X* [28]. In this sense, a compensation might be substantially non-controlling up to a certain amount. Conversely, a big difference between compensation and economic loss (*C >> L*) might constitute a substantially controlling incentive—undermining the control of the individual over her choices. One might imagine that the compensation of an average monthly wage would make it impossible for an economically struggling subject to carefully and freely consider her options and choices. Some authors fear that the incentive could even become ‘coercive’ and interfere with the subjects autonomous decision [29]. However, there is no standard account of coercion and what it involves [30].

In contrast, Wilkinson opposes the idea that vast sums of money (*enormous offer*), or financial offers made towards people in desperation (*desperate offeree*), undermine autonomy [31]. The idea here is that even though recipients of *enormous offers* and *desperate offerees* will find it hard to refuse, it does not follow that they are unable to consent validly. Otherwise, it would be impossible for anyone ever to consent validly to lifesaving treatments. Simply because a proposal is extremely attractive does not mean that it cannot be accepted voluntarily. Even more radical, Conly right out rejects the idea of autonomy as inviolable [32]. If we care about people and value their having happy, fulfilling lives, goes her argument, we must even embrace coercive paternalism in certain cases. If we agree with Conly that we need to do whatever is necessary, even if unwelcome by the beneficiary, to allow people to live the lives they truly want to live (such as one without HIV), we might worry less about a possible infringement of economic compensations on autonomy.

In any case, there does not seem to be an easy way to determine an appropriate threshold of economic compensation and this threshold would be highly variable, depending very much on the targeted individual’s financial and personal situation. Clearly, the risk of infringing on autonomy is increasing, the further we move away from C = L. In addition, ethical doubts about the efficient use of resources come into play if the compensation is disproportionately high [29].

### Justice, beneficence and non-maleficence

Besides *respect for autonomy*, *beneficence*—to act in the best interest of the patient—*non-maleficence*—to not be the cause of harm—and *justice* are classical principles of medical ethics. It has been reasoned that paying incentives might be unfair to those already behaving in a healthy way, e.g., those being already circumcised but who did not receive any compensation [33, 34]. Nevertheless, it must be acknowledged that those individuals do also benefit because raising the rate of circumcision further reduces their own (and their family’s) risk, and it reduces overall healthcare costs [7, 35]. Moreover, it has been feared that people might worsen their behaviour to become eligible for a particular incentive, for instance, gaining weight to receive an incentive for weight-loss [35]. This is apparently not a concern with VMMC.

To assure justice, the incentive should be a fair exchange: in VMMC the participant benefits from the incentive itself (net financial benefit = *C*—*L*), an evidence-based reduced risk of acquiring HIV and cancer-causing HPV, and, arguably, improved public health; whilst the programme benefits from the improvement in public health. *Beneficence*, in this context, is closely linked to the scientific evidence regarding the protective effect of VMMC, and its evaluation might, therefore, change with new studies. On the other hand, the participant suffers pain and assumes the risk of surgery, including post-surgery complications, e.g., infection. In addition, burdens may include more than pain and possible complications, since, for example, some men may get circumcised despite their ethnic identity as part of a non-circumcising tribe, causing other problems (such as the ability to marry certain partners). For instance, it was found that for Xhosa men in South Africa *ulwaluko* (the traditional circumcision without stitching) is a crucial marker of masculine identity. Contrarily, a stitched penis is considered forgery and exhibits a lack of manliness [36]. Such social and cultural impacts are hard to compensate for and add to the described difficulties in quantifying appropriate compensation. To make this exchange as fair as possible, providers have to ensure the procedure is as safe as possible (non-maleficence), including ongoing safety and quality monitoring of the program and services. The provider would also have to make sure that the incentivized procedure is on offer to all men in the targeted population [37] and that the compensation is paid for participation rather than the achievement of a specific health-related goal [38]. Thus, the compensation should only be paid for the decision to take part in a VMMC programme, regardless of the outcome (in terms of, e.g., successful surgery or HIV-prevention); otherwise, the incentive may be unfair for people who fail to achieve the desired outcome for reasons beyond their control [35, 37, 39]. It is also vital that providers disburse the full economic compensation in a reasonable time, thereby keeping their promises and commitments.

When it comes to health equity and access to healthcare, it can be argued that providing economic compensations might enable men who are economically less well-off to make a rational decision in the first place. By making them less dependent on external financial constraints, inequity in the ability to participate in the preventive measure could be reduced. Through the elimination of costs for the individual, subjects may be enabled to make individual health-related risk/benefit analyses (possibly, but not necessarily, taking public health considerations into account) with less interference by economic variables. Following the same line of argument, it can be argued that the economic loss (*L*) should be considered a hidden co-payment for VMMC—even a form of disincentive—having the most substantial effect on vulnerable populations such as the most deprived quintile. In other words, men need to ‘pay’ for the procedure with their economic loss. Obviously, the economically least well-off will be most prone to this disincentive. Some argue that for the least fortunate, the cost of prevention should be minimized and ideally neutralized to zero [35]. It has also been argued, in the context of tuberculosis therapy, that by providing persons with incentives in exchange for treatment adherence, we value the ethical principle of reciprocity, since it is appropriate for the society to provide something in return when individuals accept burdens for the benefit of the community [40]. Therefore, by enabling men to benefit from VMMC through removing a barrier which disproportionally affects the economically disadvantaged, we might increase health equity and adhere to the principle of reciprocity.

### Personal responsibility

It is feared that through the introduction of health-related rewards, peoples’ responsibility and intrinsic motivation to take care of their health and to contribute to the common good will get undermined [30, 33, 38, 39, 41, 42]. Paying men to undergo VMMC might be perceived as plain wrong by people considering VMMC the right thing to do since it means paying them to do something which they *should have done anyway*; whereas it constitutes another type of wrongness for those vehemently opposed to VMMC, paying men for something they *should not have done anyway* [30]. Another worry is that men might worsen their behaviour after having undergone VMMC, for example, decreasing their use of condoms for protection. Encouragingly, concerns of such post-operative, risk-increasing changes in behaviours are not supported by current evidence [43, 44]. It is also feared that introducing compensations might unintentionally create expectations of getting rewarded for all kinds of interventions, even non-public-health-related ones [35]. In this regard, one should be aware that the long-term effects of introducing incentives are unknown [35], and their impact in different cultural settings remains unclear. In any case, it seems vital to monitor programs involving incentives, including the postoperative period continually, and to seek inputs of local communities.

### Effectiveness, cost-effectiveness and feasibility

Interventions should be effective [37, 45] and—in combination with the economic compensation and related programme costs—cost-effective. Why should we risk infringing on other values (e. g., autonomy, personal responsibility), if compensations do not significantly increase VMMC uptake? The same might be true if we consider the effect too small to justify a tradeoff. There is robust evidence for the effectiveness of VMMC in reducing men’s risk of heterosexual acquisition of HIV-1 infection [2–5], as well as HPV [46]. In the case of VMMC, the intervention has been shown through modelling to be cost-effective, and in many countries, cost-saving [7]. To meet global HIV prevention targets, millions of circumcisions would have to be conducted—and millions of compensations paid. Thus, the cost-effectiveness needs to be demonstrated for a particular compensation scheme combined with the programme costs. In addition, a possible shift in the benefit-cost ratio, i.e., the ratio between additional benefit and additional costs of reimbursement for VMMC has to be considered. Using a larger amount to get a more significant effect might be appealing. However, it has been shown that uptake rates for HIV testing results increased only slightly with increased incentive size [47]. Similarly, there was no significant difference in VMMC uptake between a compensation of USD 8.75 and USD 15.00 [48]. The relationship between the incentive size and its effect might follow a (double) sigmoid function, making it cost-ineffective to increase the amount of the incentive close to the (first) upper limit. Therefore, it is crucial to understand the effect of different amounts of incentives, which could very well differ across populations and cultures. Furthermore, when designing programmes using economic compensations, feasibility—constraints at the personnel, health system and practical implementation level—must also be part of the equation. Trying to individually calculate economic compensations, for example, might simply not be feasible.

Finally, introducing economic compensations for VMMC at a national level will undoubtedly be a costly affair. Countries with struggling health systems and constrained public health budgets must decide whether such a programme would not take away resources from other programmes and interventions deemed more important or effective in improving population health. Even with considerable HIV incidence, there might be more pressing health issues in some countries. However, it should not be forgotten that the additional cost of incentivizing men to undergo VMMC might even be amortized in the long run—through the prevention of additional HIV infections which would have ultimately charged the health system more. While these considerations sound rather technical and economical, they nevertheless possess a strong ethical dimension. More specifically, when VMMC programmes and compensations are paid by the tax-payer or come from systems of solidarity, such as universal health coverage, complex issues of resource allocation come into play.

### Type of compensation

Although some authors have suggested that there is a difference between non-monetary and monetary incentives [48], it remains unclear why there should be a fundamental difference between these two. Non-monetary incentives, e.g. food-vouchers, can be transferred into money by the subjects by merely selling the vouchers or the objects obtained. Personalized non-monetary incentives, like, for example, non-transferable vouchers for medical treatments, e. g., dental treatments, might be an exception in this regard. However, using such incentives raises ethical questions of its own and makes quantifying the incentive and its effect on individuals even more difficult. Non-monetary incentives are more prone to changes in their value, as food prices, for example, might change rapidly and unpredictably. Conversely, monetary incentives usually have much greater value stability—unless there is a high inflation rate—and are less dependent on regional and local conditions. However, a possible difference between various types of incentives, (e.g., monetary vs non-monetary, food-vouchers vs food) in their effectiveness, at the same cost for the provider, might come into play regarding efficiency. For instance, people might be, for different reasons, more attracted to a specific non-monetary incentive than to the equivalent amount of money. In-kind-incentives, frequently used in Germany [49], e.g., exercise-equipment or heart-rate monitors, seem hardly applicable in VMMC.

### Short-term nature of rewards

The decision for VMMC is a lifelong decision that cannot be reversed. On the one hand, this emphasizes the need for a non-controlled, well-reflected choice on the part of the participant. On the other hand, it dispels a point of criticism on incentives: their short-term character [38]. It has been shown that the desired changes in habitual health-related behaviours weaken over time after removing the incentive [50]. There might even be a negative effect on intrinsic motivation, as extensive research has revealed that tangible rewards tend to have a negative effect on intrinsic motivation [51]. However, in the case of VMMC, a one-time reward can be applied to get a long-term effect [44] on individual and public health without the need to constantly and permanently change a specific behaviour. This feature of VMMC renders it also more attractive from an economic viewpoint.

### Community engagement and acceptability

As with any other public health intervention, providers should seek to build and maintain trust and strive to understand the perception of their intervention within the targeted population. Respecting other general ethical considerations of public health, such as transparency, keeping promises and commitments, as well as protecting privacy and confidentiality [45] will help to achieve trust. Providers should seek input from within the target population regarding their programmes, both before the initialization and while monitoring. Community involvement is critical, as compensations may be seen, in some communities, as inappropriate, or even insulting, i.e. attempting to “buy” the participant’s cooperation [40]. Incentives are unlikely to be effective if they are thought inappropriate, and the meaning and acceptability of the offerings must remain a mystery to programme providers without effective community engagement and feedback.

### Limitations of economic compensation interventions in VMMC

There is limited evidence that providing economic compensation can increase VMMC uptake [22]. Nevertheless, it should be emphasized that many different reasons for men not taking up VMMC have been reported, e.g., fear of pain, refusal of the partner, religious or cultural reasons, or not believing to be at personal risk of HIV [52–54]. It remains unclear, if—and to what extent—economic compensation affects these factors, and stakeholders may want to consider (improving) the usage of *rational persuasion* (and possibly *nudging*) to address them. Over the excitement that economic compensations are able to boost VMMC uptake at the population level, it must not be forgotten that they will not convince men who strongly deem VMMC unacceptable for different, non-economic reasons. In any case, a multi-layered approach to enhancing uptake will be required. On another note, it has been found that a cost reduction in VMMC is preferentially attracting men who are practising safer sex—those who are at least risk [53]; a finding that was contradicted by Thirumurthy et al. [48], showing a significant increase in the uptake of VMMC among participants at higher risk of HIV-acquisition.

### Justificatory conditions

Childress et al. propose five “justificatory conditions” to help determine whether promoting public health warrants overriding other values in particular cases, namely: effectiveness, proportionality, necessity, least infringement and public justification [45]. If we decide to infringe other general moral considerations, such as autonomy, when using incentives, we must show that by doing so, we effectively enhance public health. Although pilot studies show the effectiveness of economic compensations in VMMC, policy makers should keep in mind that these effects might differ and depend on many variables such as the cultural background. It also seems pertinent to ensure ongoing effectiveness through close monitoring of programmes. After weighing the potential public health benefits against the infringed general moral considerations, decision-makers should come to the conclusion that the positive aspects prevail—a condition termed proportionality. However, it does not follow that simply because a public health intervention is effective and proportional we should implement it. Providers must also ensure that there are no other, morally less-troubling, alternatives with the same or equal effectiveness and proportionality. Economic compensations are certainly to be preferred above more coercive measures such as making VMMC mandatory by law, but maybe there are less-troubling alternatives to achieve the goal of boosting VMMC? In the case of economic compensations, alternatives such as further rational persuasion should be considered. Furthermore, decision-makers should seek to minimize the infringement of general moral considerations. In the case of VMMC, for example, the size of the incentive might determine the infringement on autonomy. Finally, decision-makers must explain and justify their programmes’ concomitant infringements to the relevant parties, including those affected by the infringement. When using incentives in VMMC, public health agents should be transparent about their programmes, their ethical evaluation and decision making, and must not try to conceal identified risks.

## Conclusion

In this paper, we did not discuss the general ethical and human rights issues of VMMC itself—a controversy which has been addressed [23–25] and goes beyond the scope of this paper—but instead focused on the ethical implications of *paying economic compensations* to boost the uptake of VMMC.

Whenever considering the use of economic compensations for VMMC, decision-makers should be aware that a perfect reimbursement of the actual economic loss is unrealistic in heterogenous target populations (leading to the incentivization of at least parts of the population). Therefore, the ethical implications of incentives, as presented in this paper, have to be taken into account when developing, implementing or monitoring programmes that provide economic compensations for VMMC. If decision-makers wish to avoid the pitfalls of incentives as much as possible, they should try to approximate the compensations being paid to the actual economic loss to the greatest extent possible (C ≊ L)—a task that might prove to be arduous.

However, there might be good reasons to pay more—deliberately utilizing the additional benefit of incentivizing men. As we have seen, by doing so, we might infringe on other ethical principles or general moral considerations. Therefore, public health agents should carefully evaluate to what extent their particular programme does so—and if the goal of promoting public health through the use of incentives warrants overriding other values. The five “justificatory conditions” by Childress et al. might provide a reasonable framework and starting point in this regard. It is not possible to give a final conclusion here—in general favour for or against the use of economic compensations in VMMC—as the balance might tip to one or the other side depending on the particular programme and many variables, such as the size of the compensation paid, the targeted population, and the current scientific evidence on HIV prevention effectiveness of VMMC, amongst others.

In any case, involving the targeted communities before implementing any compensation programmes for VMMC seems indispensable. Not only will it enable policymakers to understand better how such compensation is perceived by the targeted population, but honest efforts to listen and respond to people’s concerns will also build trust and credibility. Programmes using economic compensation in VMMC should ensure sustained and close monitoring due to very little experience in this domain and since their impact might significantly differ across populations, societies and cultures. Public health agents and their institutions must be willing and prepared to modify or cease their programmes quickly should the adverse effects of financial compensation prevail.
